# Simple sequence repeat markers that identify *Claviceps* species and strains

**DOI:** 10.1186/s40694-016-0019-5

**Published:** 2016-01-15

**Authors:** Barbara S. Gilmore, Stephen C. Alderman, Brian J. Knaus, Nahla V. Bassil, Ruth C. Martin, James E. Dombrowski, Jeremiah K. S. Dung

**Affiliations:** 1 USDA ARS Forage Seed and Cereal Research, 3450 SW Campus Way, Corvallis, OR 97331 USA; 2 USDA ARS Horticultural Crops Research Unit, 3420 NW Orchard Ave, Corvallis, OR 97331 USA; 3 USDA ARS National Clonal Germplasm Repository, 33447 Peoria Road, Corvallis, OR 97333 USA; 4 Oregon State University, Central Oregon Agricultural Research Center, 850 NW Dogwood Lane, Madras, OR 97741 USA

**Keywords:** *Claviceps purpurea*, *Claviceps humidiphila*, ERGOT, Simple sequence repeat primers, Perennial ryegrass, Kentucky bluegrass

## Abstract

**Background:**

*Claviceps purpurea* is a pathogen that infects most members of Pooideae, a subfamily of Poaceae, and causes ergot, a floral disease in which the ovary is replaced with a sclerotium. When the ergot body is accidently consumed by either man or animal in high enough quantities, there is extreme pain, limb loss and sometimes death.

**Results:**

This study was initiated to develop simple sequence repeat (SSRs) markers for rapid identification of *C. purpurea*. SSRs were designed from sequence data stored at the National Center for Biotechnology Information database. The study consisted of 74 ergot isolates, from four different host species, *Lolium perenne*, *Poa pratensis*, *Bromus inermis*, and *Secale cereale* plus three additional *Claviceps* species, *C. pusilla*, *C. paspali* and *C.* *fusiformis.* Samples were collected from six different counties in Oregon and Washington over a 5-year period. Thirty-four SSR markers were selected, which enabled the differentiation of each isolate from one another based solely on their molecular fingerprints. Discriminant analysis of principle components was used to identify four isolate groups, CA Group 1, 2, 3, and 4, for subsequent cluster and molecular variance analyses. CA Group 1 consisting of eight isolates from the host species *P. pratensis*, was separated on the cluster analysis plot from the remaining three groups and this group was later identified as *C. humidiphila*. The other three groups were distinct from one another, but closely related. These three groups contained samples from all four of the host species. These SSRs are simple to use, reliable and allowed clear differentiation of *C. humidiphila* from *C. purpurea*. Isolates from the three separate species, *C. pusilla*, *C. paspali* and *C.* *fusiformis*, also amplified with these markers.

**Conclusions:**

The SSR markers developed in this study will be helpful in defining the population structure and genetics of *Claviceps* strains. They will also provide valuable tools for plant breeders needing to identify resistance in crops or for researchers examining fungal movements across environments.

## Background

Mild winters and warm, dry summers in the Pacific Northwest of North America, where over 60 % of the world’s cool season grass seed is produced [1], are ideal for grass seed production. In Oregon, perennial ryegrass seed, *Lolium perenne*, and Kentucky bluegrass seed, *Poa pratensis*, are important crops. In 2014, 85 million kilograms of perennial ryegrass seed were produced, with a farm gate value of US$ 158 million [2]. Kentucky bluegrass seed produced in 2014 was over 8 million kilograms and valued at almost US$ 23 million [2]. *Claviceps* species are important pathogens in the Pacific Northwest, where *Claviceps* infection can reduce yields in Kentucky bluegrass by as much as 47 % by weight [3]. The genus *Claviceps* includes about 45 species [4] characterized by sclerotia which germinate to produce stalked, spherical fruiting bodies (capitula) embedded with perithecia. Ascospores produced within and ejected from the perithecia infect ovaries of grasses and sedges, transforming each infected ovary into an elongated or spherical sclerotium, depending on the *Claviceps* species. The best known species is *C. purpurea* which infects hundreds of grasses, primarily in the Pooideae subfamily of the grass family, including wheat, rye, and barley [5]. Alkaloids produced in the sclerotia are toxic, and if ingested by animals or humans result in ergotism. Ergotism is a condition characterized by constriction of the blood vessels, and can give rise to abortion, gangrenous limbs, and death from continued ingestion of the alkaloids [5].

Sclerotia of *C. purpurea* are black to purple-black and often extend beyond the host lemma and palea. Size of the sclerotium is proportionate to host seed size, with sclerotium size increasing with size of the host seed [5]. In addition to ascospores, conidia are produced during the early stages of infection and can contribute to secondary spread of the pathogen. Conidia mix with plant sap and ooze from infected florets in what is commonly referred to as the honeydew stage of disease development [5].

Breeding for resistance to *C. purpurea* or *C. humidiphila* requires an efficient means of identifying *Claviceps* strains, and improved knowledge of resistance genes in crops such as perennial ryegrass and Kentucky bluegrass [6–8]. The ability to fingerprint *C. purpurea* isolates would allow identification of more virulent strains and assist plant breeders in the evaluation of the resistance genes that are available in the different cereal or grass gene pools. In 1997, Jungehülsing and Tudzynski determined that non-rye ergot isolates were less aggressive on rye than isolates from rye [9]. Pažoutová [10] also concluded that the genetic resistance of host grasses may vary with the *C. purpurea* isolate used for evaluation. Therefore, the isolate strains should originate from locations that are similar to those intended for growth of the resistant cultivar [10]. This suggests that an appropriate isolate-host pair is required for identifying resistance in host crops. Isolate identification would also allow monitoring fungal movement in terms of both distance and speed across previously non-infected fields. SSR markers would then prove to be a valuable molecular tool for differentiating *Claviceps* species and strains for breeding programs and for gaining a better understanding of *Claviceps* population biology and genetics.

Previously, sixteen Randomly Amplified Polymorphic DNA (RAPD) primer pairs were successfully used to discriminate between 29 field isolates of *C. purpurea* from various parts of Europe [9]. In addition to RAPDs, alkaloid analysis and conidial measurements were also used to discriminate *C. purpurea* isolated from or found growing in three diverse environments: open meadows and fields, shady or wet grassy areas, and salt marshes [11, 12]. These three populations were originally designated as genotypes G1, G2 and G3 respectively but are now recognized as *C. purpurea* sensu stricto, *C. humidiphila*, and *C. spartinae*, respectively. The sclerotia of the three species were found to produce different sets of alkaloids; *C. purpurea* sensu stricto sclerotia contains ergotamine, ergosine, ergocornine, ergocryptine and ergocristine; *C. humidiphila* produces ergosine, ergocristine and ergocryptine; and *C. spartinae* produced ergocristine and ergocryptine [11, 13]. In 2015, a new genetic group was added, G2a (G4), *C. arundinis*, which inhabited hosts found growing in very wet areas and produced sclerotia that contained the alkaloids, ergosine, ergocristine, ergocristam and ergosedmam [13, 14].

Amplified Fragment Length Polymorphism (AFLP) marker patterns and *Eco*RI restriction site polymorphism in the 5.85S ribosomal DNA (rDNA) have also been used [11, 15]. Based on RAPD and AFLP analyses of *Spartina alterniflora* ergot, G1, G2, and G3 isolates were each present in a specific geographical area, but G3 had little variation, possibly indicating recent introduction [15]. Phylogenetic analyses of *C. purpurea* populations using DNA sequences from an internal transcribed spacer region (ITS) and a portion of the gene encoding β-tubulin indicated that the G1 types had diverted significantly from the G2/G3 types [16]. According to Pažoutová [11], these populations can only be differentiated by molecular methods, and are not phenotypically, host or habitat distinguishable, but do appear to be habitat-specialized.

Additional molecular resources for *C. purpurea* are available and include expressed sequence tags (ESTs) [17], and a genome sequence, based on Roche/454 chemistry [18]. While informative techniques have been used to differentiate *C. purpurea* isolates, SSRs were not developed despite their reported value as co-dominant, multi-allelic, abundant, easily implemented, and highly reproducible markers [19]. Existing sequence resources can be applied for the development of additional SSR markers in a genus like *Claviceps* that has few available SSRs. Given the importance of identifying different isolates, the objective of this study was to develop SSR markers to facilitate differentiation of *Claviceps purpurea* isolates and its closely related species.

## Results and discussion

Twenty-five of the 74 isolates used in this study were previously characterized by RAPD patterns, ITS sequences and mating type PCR assays by Scott et al. [20] and identified as *C. purpurea*, (G1), isolated from *L. perenne* and *P. pratensis, and C. humidiphila* (G2), from *P. pratensis*. Only molecular methods were used to distinguish these isolates from each other, since they were collected from similar environments. The SSRs developed in this study were evaluated in the 74 isolates.

### Simple sequence repeats (SSRs)

The microsatellites were mined from 12 assembled genomic sequences from data stored at NCBI [21]. Primers were designed for 267 SSR sequences but only the first 192 primer pairs were evaluated in this study. In the first screening, 123 of the 192 primers tested in four isolates (Cp03, Cp26, Cp32 and Cp33) (Table 1) appeared polymorphic, 20 had missing data or null alleles, 17 were monomorphic and 32 failed to amplify. Eight isolates (Cp25, Cp26, Cp27, Cp29, Cp30, Cp31, Cp32, and Cp33) (Table 1) were used in the second screening to evaluate 59 primer pairs that were selected based on polymorphism in the first screening and ease of multiplexing. The criteria for primer selection of the resulting fingerprinting panel were ease of scoring, high polymorphism and amplicon sizes that permitted multiplexing. The results from the second screening yielded 34 primer pairs that were placed into seven multiplex pools post PCR amplification for capillary electrophoresis separation.

**Table 1:** List of 74 isolates of *Claviceps purpurea* (Cp) and one each of *C. pusilla, C. paspali* and *C. fusiformis*

Sample	Host	Year	Cultivar	State	Cty	CA Group
*C. purpurea*, Cp01	*L. perenne*	2012	Pavilion	OR	Um	2
*C. purpurea*, Cp02	*L. perenne*	2012	Pavilion	OR	Um	3
*C. purpurea*, Cp03^a^	*L. perenne*	2012	Pavilion	OR	Um	3
*C. purpurea*, Cp04	*L. perenne*	2012	Provocative	OR	Um	2
*C. purpurea*, Cp05	*L. perenne*	2012	Provocative	OR	Um	2
*C. purpurea*, Cp06	*L. perenne*	2012	Top Hat II	OR	Um	4
*C. purpurea*, Cp07	*L. perenne*	2012	Top Hat II	OR	Um	3
*C. purpurea*, Cp08	*L. perenne*	2012	Pavilion	OR	Um	2
*C. purpurea*, Cp10	*L. perenne*	2012	Pavilion	OR	Um	3
*C. purpurea*, Cp11	*L. perenne*	2011	Esquire	OR	Um	3
*C. purpurea*, Cp12	*L. perenne*	2011	Esquire	OR	Um	3
*C. purpurea*, Cp13	*L. perenne*	2011	Esquire	OR	Um	3
*C. purpurea*, Cp14	*P. pratensis*	2012	Midnight	WA	Be	1
*C. purpurea*, Cp15	*P. pratensis*	2012	Midnight	WA	Be	1
*C. purpurea*, Cp16	*P. pratensis*	2012	Midnight	WA	Be	1
*C. purpurea*, Cp20	*P. pratensis*	2012	Seed screenings	WA	Be	1
*C. purpurea*, Cp21	*P. pratensis*	2012	Seed screenings	WA	Be	1
*C. purpurea*, Cp22	*P. pratensis*	2012	Arrowhead	WA	Be	1
*C. purpurea*, Cp23	*P. pratensis*	2012	Arrowhead	WA	Be	1
*C. purpurea*, Cp25^b^	*S. cereale*	2012	Wild	OR	Um	3
*C. purpurea*, Cp26^a,b^	*L. perenne*	2011	Seed screenings	OR	Um	3
*C. purpurea*, Cp27^b^	*L. perenne*	2011	Seed screenings	OR	Um	4
*C. purpurea*, Cp29^b^	*L. perenne*	2012	Esquire	OR	Um	4
*C. purpurea*, Cp30^b^	*B. inermis*	2011	Wild	WA	Wh	2
*C. purpurea*, Cp31^b^	*B. inermis*	2011	Wild	WA	Wh	2
*C. purpurea*, Cp32^a,b^	*P. pratensis*	2013	Seed screenings	OR	Un	1
*C. purpurea*, Cp33^a,b^	*P. pratensis*	2013	Baron	OR	Un	2
*C. purpurea*, Cp34	*L. perenne*	2013	Provocative	OR	Um	3
*C. purpurea*, Cp35	*L. perenne*	2013	Provocative	OR	Um	3
*C. purpurea*, Cp37	*L. perenne*	2013	Zoom	OR	Um	3
*C. purpurea*, Cp38	*L. perenne*	2013	Zoom	OR	Um	3
*C. purpurea*, Cp39	*L. perenne*	2013	Zoom	OR	Um	2
*C. purpurea*, Cp40	*L. perenne*	2014		OR	Um	4
*C. purpurea*, Cp41	*L. perenne*	2014		OR	Um	3
*C. purpurea*, Cp42	*L. perenne*	2014		OR	Um	3
*C. purpurea*, Cp43	*L. perenne*	2014	Casper	OR	Um	3
*C. purpurea*, Cp44	*L. perenne*	2014	Casper	OR	Um	4
*C. purpurea*, Cp45	*L. perenne*	2014	Casper	OR	Um	4
*C. purpurea*, Cp46	*L. perenne*	2014	Esquire	OR	Um	4
*C. purpurea*, Cp47	*L. perenne*	2014	Esquire	OR	Um	4
*C. purpurea*, Cp48	*L. perenne*	2014	Esquire	OR	Um	4
*C. purpurea*, Cp49	*L. perenne*	2014	Frontier	OR	Um	3
*C. purpurea*, Cp50	*L. perenne*	2014	Frontier	OR	Um	3
*C. purpurea*, Cp51	*L. perenne*	2014	Frontier	OR	Um	4
*C. purpurea*, Cp52	*P. pratensis*	2010	Seed screenings	OR	Je	2
*C. purpurea*, Cp53	*P. pratensis*	2010	Seed screenings	OR	Je	2
*C. purpurea*, Cp54	*P. pratensis*	2010	Seed screenings	OR	Je	2
*C. purpurea*, Cp55	*L. perenne*	2013	Esquire	OR	Be	4
*C. purpurea*, Cp56	*L. perenne*	2013	Esquire	OR	Be	3
*C. purpurea*, Cp57	*L. perenne*	2013	Esquire	OR	Be	4
*C. purpurea*, Cp58	*L. perenne*	2014		OR	Um	3
*C. purpurea*, Cp59	*L. perenne*	2014		OR	Um	4
*C. purpurea*, Cp61	*L. perenne*	2013	Zoom	OR	Um	3
*C. purpurea*, Cp62	*L. perenne*	2013	Zoom	OR	Um	3
*C. purpurea*, Cp63	*L. perenne*	2013	Zoom	OR	Um	3
*C. purpurea*, Cp64	*L. perenne*	2013	PST-2M20	OR	Be	4
*C. purpurea*, Cp65	*P. pratensis*	2012	Baron	OR	Un	2
*C. purpurea*, Cp66	*L. perenne*	2012	seed screenings	OR	Um	4
*C. purpurea*, Cp67	*L. perenne*	2012	seed screenings	OR	Um	4
*C. purpurea*, Cp69	*L. perenne*	2014	Pavillion	OR	Um	4
*C. purpurea*, Cp70	*L. perenne*	2014	Pavillion	OR	Um	3
*C. purpurea*, Cp71	*L. perenne*	2014	Pavillion	OR	Um	3
*C. purpurea*, Cp72	*L. perenne*	2013	Provocative	OR	Um	4
*C. purpurea*, Cp73	*L. perenne*	2013	Provocative	OR	Um	3
*C. purpurea*, Cp74	*L. perenne*	2013	Provocative	OR	Um	3
*C. purpurea*, Cp78	*P. pratensis*	2012	Baron	OR	Un	2
*C. purpurea*, Cp79	*P. pratensis*	2012	Baron	OR	Un	2
*C. purpurea*, Cp80	*P. pratensis*	2012	Baron	OR	Un	2
*C. purpurea*, 14-036-F	*L. perenne*	2013	Pavillion	OR	Um	4
*C. purpurea*, 14-037-F	*L. perenne*	2013	Pavillion	OR	Um	4
*C. purpurea*, 14-038-F	*L. perenne*	2013	Pavillion	OR	Um	4
*C. purpurea*, 14-039-F	*L. perenne*	2013	Pavillion	OR	Um	4
*C. purpurea*, 14-040-F	*L. perenne*	2013	Pavillion	OR	Um	4
*C. purpurea*, 14-041-F	*L. perenne*	2013	Pavillion	OR	Um	3
*C. pusilla*						
*C. paspali*						
*C. fusiformis*						

Host plant species, year of collection, name of host cultivar, state where collected, county (Cty) and cluster analysis (CA) Group are provided

ST State where sample was collected, OR (Oregon), WA (Washington)

Cty County where sample was collected, Be (Benton), Je (Jefferson), Um (Umatilla), Un (Union), and Wh (Whitman)

^a^Four samples used for the first screening

^b^Eight samples used for the second screening

### Fingerprinting and genetic diversity evaluation

The motifs for the 34 SSRs included two dinucleotides (5.9 %), 22 trinucleotides (64.7 %), six tetranucleotides (17.6 %), and four pentanucleotides (11.7 %) (Table 2). The number of alleles in the 77 evaluated isolates varied from a low of two alleles for primers Cpur20 and Cpur72 to a high of 15 alleles for Cpur56, and averaged 5.8 alleles per primer pair (Table 2). Twenty-one SSR primer pairs generated one allele per sample, while 12 primer pairs yielded up to two alleles for some or all samples, and one primer pair, Cpur69, amplified three alleles in one individual, 14–040 (Table 2).

**Table 2:** List of 34 SSRs evaluated in 74 isolates of *C. purpurea* and three related species (*C. pusilla, C. paspali*, and *C. fusiformis*)

Primer	Primer sequence	SSR motif	Allele range (bp)	*A*	*H*	Alleles/isolate	Alleles for four species			
							*C. humidiphila*	*C. pusilla*	*C. paspali*	*C. fusiformis*
Cpur2^a,b^	F: GCTGGCTTCGTGTTCACAG	(AAAC)_4_	443–466	7	1.56	1		191, 236	138, 240	240
	R: GGAAACCTCGTTGCTGACC									
Cpur6^a^	F: GACTGGCATCCGCATTTCC	(ATGT)_6_	317–375	7	1.34	2		101, 243	103, 245, 442	NA
	R: GTCGCGCGTGAATCTTGAG									
Cpur7	F: TCAACGCACAGAGCAATCC	(AGC)_5_	371–395	9	1.67	1	351	243, 386	156, 427	432
	R: CTGCACAAGCACTGGAAGG									
Cpur8^b^	F: TTCTCCCGCCGTATAACCG	(GGT)_8_	302–317	7	1.62	1	295	97, 203	97, 136	127
	R: GTTCGCGATCTGACGTTCC									
Cpur12	F: CGGAGCAAATGTTCGTCCC	(GAT)_4_	413–433	4	1.15	1	419	102,125	107, 247, 364, 371	231, 378
	R: AGTACATCGGCCTGGAACC									
Cpur14	F: GCGCCTGGCATAATAGTGG	(ACAT)_6_	461–481	7	0.69	1	425	203, 244	135, 185	407, 432
	R: TCGAAGTCGAGAGGAACCG									
Cpur20	F: GACACCCATTGGCAACCAG	(ACC)_4_	318–321	2	0.42	1		159, 441	203, 353, 502	344
	R: TCAGAGGCGCAGTATCGAC									
Cpur23^a^	F: TGCCTTGCCTTCTTTCAGC	(ACAT)_5_	350–365	5	1.08	2	601	123, 166, 211, 378	151, 228, 341	246, 412, 477
	R: GGCAACTTGGCAGAAGACC									
Cpur24	F: CCGATTGAGCAACAGCTCG	(AAG)_7_	404–438	7	0.75	1	409	204, 220, 241, 368	102, 210	190, 270, 289, 354
	R: CTGAGCGGCAAAGTCATCC									
Cpur26	F: GGTCGTCTATGGCGTGGAG	(GGGAT)_4_	411–426	4	0.81	1		164, 496	92, 301, 421	84, 204, 251
	R: CTCCGAATCAATCCCGTGC									
Cpur30^a,b^	F: CCACCGGGCATTGTTGAAG	(ACATT)_4_	225–305	6	1.03	2	265	160	166, 247, 396, 417	160, 321, 506
	R: CATGTCTCAAGGCGGCAAG									
Cpur31^b^	F: ACTCCCGCTCAATAAGCCTC	(CTT)_4_	233–245	7	1.25	2	103, 163	295	233	203
	R: CAGAATATGCAAAGGGTGCG									
Cpur32^a,b^	F: AACGCAGCGCATGAAAGAC	(AAC)_5_	154–182, 393–405	6	1.39	2		112, 396	176	145, 159, 176, 483
	R: TCGTGGAGTCCGTGAATGG									
Cpur34^a,b^	F: TGGTCTCGCGGTATTAGGC	(GCT)_5_	285–294	4	1.07	1		251	123, 471	NA
	R: TCTACCTTTCCGAGCCAGC									
Cpur35^b^	F: CATCGCAATGCCGTCCTAC	(CGG)_4_	369–390	5	0.97	2		112, 157, 163	163	154, 176
	R: GAACAGCCTACAGCATCGC									
Cpur40^a^	F: CCACCACAGTTGCTCTTGC	(AGC)_5_	411–417	4	1.08	1	421	136, 171	129, 233	233
	R: ACGACATGACCAGCTACCG									
Cpur41	F: ACTTGACGGCTGGGTATGG	(AC)_9_	431–454	5	1.22	1		113, 450	220, 254, 379, 500	89, 112, 376, 450
	R: GCTGTTTCCAAGACGGCAG									
Cpur43	F: TGAGTCGTGACCCAACCAG	(AAGAC)_4_	250–280	5	1.35	1		576	122, 364, 479	150
	R: TCACCCGTAAGTGTGCTCC									
Cpur48^a^	F: TCCATCCGACAACGAGCTG	(CGT)_7_	431–442	4	0.44	2		210, 267, 336	252, 358, 510	101, 266, 478, 498
	R: GGTATGCCGGAGGGTATGG									
Cpur50^a^	F: TTCCCTCGGTGACGAATCC	(CGG)_6_	321–330	4	1.58	1		147, 238	110, 231	229
	R: TCTCGGCCCTCCATCAAAG									
Cpur52^a^	F: CTCGCCATAGCAAACAGCG	(CTT)_4_	433-445	4	1.06	1		154	154, 193	127, 154
	R: CAGTACGCAGATTTGGCGG									
Cpur53^a^	F: CGATGGCCAAACTCTACGG	(CT)_6_	469–479	5	1.12	1		238	166, 471	103, 107, 471
	R: AGACACCCTGTTTGAGCCC									
Cpur55^a^	F: TTCCAAGCCTGTCGTCCTG	(CCT)_6_	472–475	3	0.71	1	469	161, 294	399	197, 441, 472
	R: GACCTGTTTGCCGACATCC									
Cpur56^a^	F: CAAAGCAGCCCGTCACTTC	(ATC)_8_	340–401	15	1.85	2	407–413	361, 375	229, 305	115, 288, 375
	R: CGCGCATTTCTGGTAGAGC									
Cpur58^a^	F: AGGTTGGACTTGGTAGGCG	(CGT)_4_	437–458	6	1.11	2	446, 449	203, 254, 290	106, 205, 319	109, 207
	R: GCTTCAGTACAGCATGGGC									
Cpur61	F: ACGGAAAGGATGGAAGCCC	(GAGT)_5_	136, 447–465	6	0.72	1		NA	90	90
	R: CATGCCAATCCCGCAGAAC									
Cpur68^a^	F: TTGTTAACGTCGCGAAGGC	(CGT)_4_	97, 255–263	3	0.50	1		252, 322	98, 146, 175, 240	139, 150, 366, 431
	R: AAGTTGGCGTTGAATGGGC									
Cpur69	F: ATTCCTCGCCCTCTTTGGG	(GGT)_8_	104, 427–436	7	0.72	3	98, 325	98, 141, 261, 468	159, 176, 325	103, 207, 408
	R: CGTCAACTTCGCCGATTCC									
Cpur72	F: AGTCGTGGGAGATTGGAGC	(AAG)_4_	275	2	0.34	1	278	485	252, 275	222, 275
	R: TCCTGTACTTGCCGAACCG									
Cpur145	F: CTGTCGCGTGCTTTCGTAG	(GAT)_5_	231–241	5	1.03	2	247	111, 200, 237	237	130, 237
	R: CAGCGCGTCTATTATGCGG									
Cpur156^a^	F: TGGCTACGGTCCTGGTTTC	(AAAGC)_4_	360–475	8	1.75	1		105, 120, 154, 522	134	107, 135, 259
	R: CCCTGCATAGAGGGTACAGC									
Cpur157	F: GCCGTGAAGTGACGAATGC	(AAG)_5_	372–405	11	1.66	2	534	253, 399	249, 327	207, 248, 381, 396
	R: AGTGTCAAGTGGGCGGTAG									
Cpur177^a^	F: CCCTCACGGTACGAGATCC	(CCG)_6_	465–475	3	0.95	1		100, 227, 312, 475	261, 317, 475	160, 317
	R: CTGCCCATCATCAAAGCCC									
Cpur192^a^	F: CGCTTTGGACCGCATGTAG	(TATG)_4_	101–110, 290–305	7	1.04	2		261, 269, 290, 441	105, 343	105, 343
	R: AGTACCTGGGCAAAGTCCG									
Ave.								5.8	1.09	1.4

SSR primer sequences, forward (F) and reverse (R), SSR motif, allele size range (bp), alleles per primer pair (*A*), Shannon-Weiner index coefficient for each primer pair (*H*), and number of alleles per isolate are listed

Unique alleles for each of the four species, *C. humidiphila, C. pusilla, C. paspali* and *C. fusiformis,* are also recorded. A blank indicates absence of unique alleles for that species while NA refers to no amplification

^a^Primers that were polymorphic in *C. humidiphila*

^**b**^Primers that distinguished isolates of CA Group 3 from those of CA Group 4

The average genetic distance between samples of *C. fusiformis*, *C. paspali*, *C. pusilla* and the remaining samples was 0.784, 0.691, and 0.767, respectively. These relatively large genetic distances indicated that samples from these three species, *C.* *fusiformis, C. paspali* and *C. pusilla,* were not closely related to the other samples. Furthermore, these species had a large number of unique alleles at many tested SSRs that were not shared with samples from the other species, supporting their distinctness (Table 1). Consequently, these three species were not included in subsequent statistical analyses.

### Statistical analysis

In order to infer and distinguish an optimal number of groups within the isolates evaluated, discriminant analysis of principle components was performed. K-means clustering was performed on 40 principle components derived from the data. For group sizes ranging from 1 to 40 groups, we examined the Bayesian information criteria (BIC) for each grouping as a function of the number of clusters, and concluded that a group number of four minimized the BIC. These four clusters were referred to with the CA prefix for cluster analysis to indicate the groups that were selected for subsequent cluster and molecular variance analyses. CA Group 1 was comprised of samples collected from the host, *P. pratensis,* in Washington and Oregon. These samples were identified as *C. humidiphila* by Scott et al. [20]. CA Group 2 consisted primarily of eight samples from the *P. pratensis* host, but also contained two samples from *Bromus inermis* and five from *L. perenne*. CA Group 3 and CA Group 4 were solely made up of samples from *L. perenne*, except for a single sample of *S. cereale* that was found in CA Group 3 (Table 1).

**Fig. 1:**
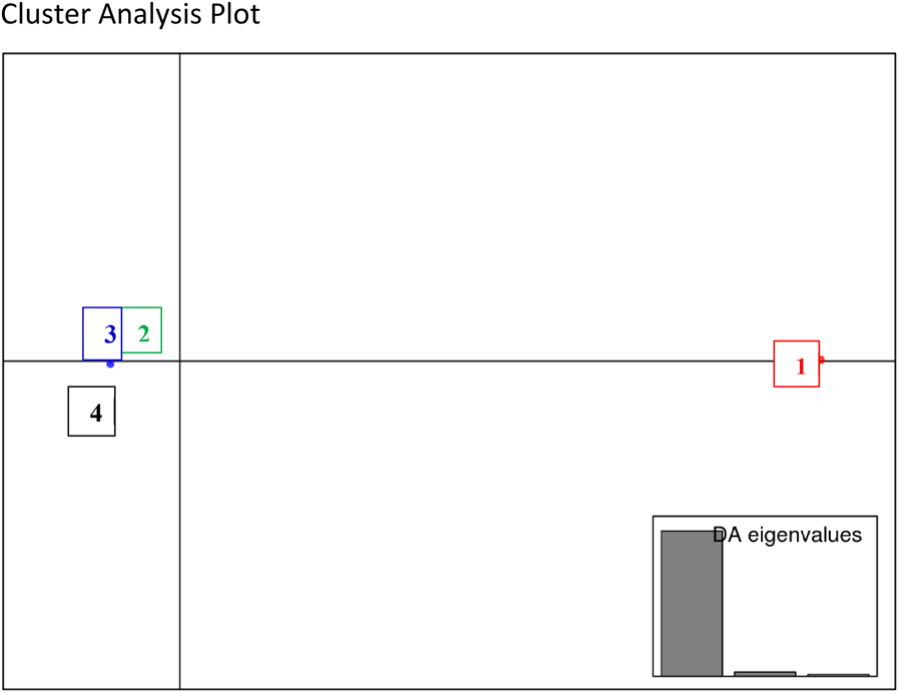
Cluster analysis plot. A plot showing the spatial relationship of our four designated CA Groups. CA Group *1* is *C. humidiphila* and CA Groups *3* and *4* are *C. purpurea*

The shared allele distances were estimates of genetic distances. The average genetic distance of CA Group 1 to the remaining 66 samples was large at 0.915. The average genetic distance for CA Group 2, Group 3 and, Group 4 were intermediate at 0.522, 0.502, and 0.495, respectively. These values supported the K-means cluster analysis results (Fig. 1). It is interesting to note that most of the Oregon samples isolated from the *P. pratensis* host were not far removed from the isolates found on the *L. perenne* host.

The CA Group 1 samples were monomorphic at 16 of the 34 SSRs, but polymorphic in the remaining 18 SSRs (Table 2). However, 13 of the 34 SSRs (Cpur07, Cpur08, Cpur12, Cpur14, Cpur23, Cpur24, Cpur30, Cpur31, Cpur35, Cpur69, Cpur72, Cpur145, and Cpur157) contained CA Group 1-specific alleles from *P. pratensis* grown in Washington that separated isolates of this group from those of the remaining three CA groups. Samples from CA Group 2, 3, and 4 were more diverse than those from Group 1 and had fewer monomorphic SSRs at 4, 5, and 6 SSRs, respectively.

Surprisingly, samples from CA Groups 3 and 4 separated into two clusters despite sharing the same host, and similar geographical origin in two counties in Oregon. All five collection years, 2010 through 2014, were represented in both groups, as were most of the cultivars (Table 1). Upon further examination of the SSR data we found that samples from these two groups had distinct alleles at seven SSRs: Cpur2, Cpur8, Cpur30, Cpur31, Cpur32, Cpur34, and Cpur35. Cluster analysis based on data for these 7 markers separated the samples into two groups. CA Group 3 and CA Group 4 did not intermingle except for one sample of CA Group 3 (Cp38) that grouped with the samples from CA Group 4 (not shown). These seven SSRs can therefore be used for distinguishing samples from each of these two groups isolated from very similar environments and hosts (Table 2).

Diversity estimates calculated at each SSR in these 74 samples consisted of the number of alleles per primer pair (*A*) and Shannon-Wiener’s index coefficient (*H*) (Table 2). The number of alleles per primer pair ranged from a low of two at Cpur20 and Cpur72 to a high of 15 alleles at Cpur56 and averaged 5.75. This was higher than expected since many fungi are very homogeneous with little polymorphism [22]. The coefficient *H* was used to measure the allelic diversity at each locus. Due to the low number of alleles at each SSR Shannon-Wiener’s index coefficient *(H)* values were low for most of the primer pairs and ranged from 0.34 at primer Cpur72 to a high of 1.85 at Cpur56 with an average of 1.09.

Sample size was more balanced in the four CA Groups than in the plant hosts *P. pratensis, L. perenne, B. inermis* and *S. cereale*. Therefore, the Analysis of Molecular Variance, AMOVA, (Table 3), was based on the four CA Groups using 147 (n−1) degrees of freedom (*df*) to code the data as diploid in the 74 samples. Where a single allele was amplified, the sample was considered homozygous at this locus. The AMOVA confirmed that the molecular variance between the samples in the groups, 56.7 %, was higher than the variance between the CA Groups, 41.5 % (Table 3).

**Table 3:** The AMOVA values

Source of variation	*df*	Sum of squares	Mean square	Expected mean square	% Variance	*P *value
Between groups	3	408.218	136.073	3.61	41.5	0.001
Between samples within groups	70	701.762	10.025	4.93	56.71	0.001
Within samples	74	11.500	0.155	0.16	1.79	0.001
Total	147	1121.48	7.63	8.7	100	0.001

The AMOVA table is based on an analysis of the groups which resulted from the cluster analysis. Presented p-values are based on one thousand permutations

**Fig. 2:**
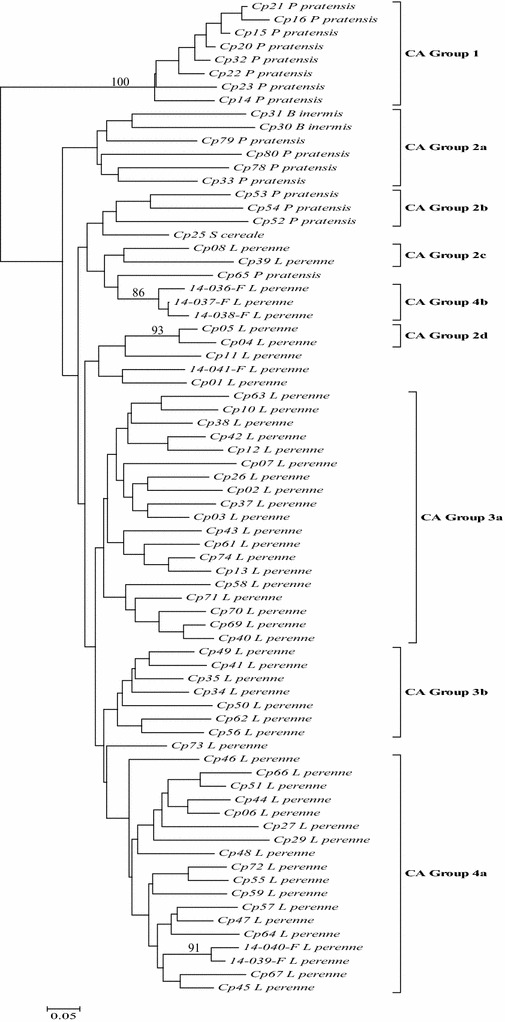
Dendrogram of 74 samples. Shared Allele Neighbor Joining dendrogram of 74 isolates. Bootstrap support of 85 or greater is indicated

As illustrated in the dendrogram (Fig. 2), each of the 74 samples produced a unique fingerprint. Samples from the Washington *P. pratensis* CA Group 1 were isolated from all other branches of the dendrogram with a high bootstrap value of 100 %. High bootstrap support of 93 % was observed in only Cp05 and Cp04 of CA Group 2d samples consisting of *P. pratensis, B. inermis,* and *L. perenne,* from Oregon. The remaining isolates of CA Group 2 were found in three branches, CA Group 2a, Group 2b and Group 2c. Isolates of CA Group 3 clustered mainly into two main branches, CA Group 2a and Group 3b, except for Cp25 from *S. cereale* that was grouped with CA Group 2b samples. A small number of isolates from CA Group 4 clustered together with high bootstrap support (86 %) in CA Group 4a while the majority were grouped together into one branch, CA Group 4b (Fig. 2).

## Conclusion

Using 34 microsatellites, we identified each of the 74 isolates of *C. purpurea* collected from four hosts and separated them into four separate groups. CA Group 1 isolates contained all the isolates from the *P. pratensis* host from Washington and were widely separated from isolates in the remaining three groups. Based on Scott et al. [20], this group represents *C. humidiphila*. Isolates from the same *P. pratensis* host but from the state of Oregon however grouped separately into CA Group 2 with *C. purpurea* samples from *B. inermis* and *L. perenne*. More work needs to be completed to evaluate the relationship between isolates of *P. pratensis* from Oregon that were in CA Group 2 and those ‘*C. humidiphila*’ isolates of CA Group 1 to determine if these two groups are both *C. humidiphila,* or if only CA Group 1 is that species.

*Claviceps purpurea* in both CA Group 3 and CA Group 4 were isolated from a single host species, *L. perenne,* and from the same geographical regions. These SSR markers easily separated the isolates into two groups, possibly indicating distinct populations. These results support the finding of Pažoutová et al. [11] that molecular are necessary to distinguish separate *Claviceps* populations. Multiple populations could arise through different seed sources, or contamination during seed harvest, storage, and cleaning. Contamination can also occur from susceptible weed grasses within fields. This indicates that proper isolate identification is critical in a breeding program aimed at developing resistance to this pathogen in a particular plant species. Repeated testing with the same isolates is necessary to identify effective resistance genes and can now be accomplished. The putative resistant genes can be tested against the same virulent strains, and resistance can then be introgressed into new cultivars. It will also be possible to follow *C. purpurea* and *C. humidiphila* movements through geographical areas.

In addition to distinguishing *C. purpurea* isolates, these SSRs also amplified in isolates from three other *Claviceps* species, *C. pusilla*, *C. paspali* and *C.* *fusiformis,* indicating possible usefulness in identifying isolates across this genus. Therefore, these markers may also be valuable for species identification.

## Methods

### Fungal cultures and DNA extraction

The 74 isolates used in this study were collected from *P. pratensis* and *L. perenne* in Oregon and Washington over a period of 5 years (2010–2014). We use the prefix Cp to refer to each *C. purpurea* isolate.

Sclerotia were surface sterilized by dipping in 95 % ethanol for 30 s, soaking in 0.6 % sodium hypochlorite solution for 1 min, and rinsing in sterile water for 15 s. Sclerotia were bisected with a flame-sterilized blade and placed, cut surface down on water agar in a 9.5 cm diameter petri plate. Single hyphal tips were each transferred to potato dextrose agar to establish hyphal tip cultures.

Cultures used for extraction were raised on a *Claviceps* medium, containing potato dextrose agar (PDA) (18 g), yeast extract (1 g), malt extract (5 g), sucrose (5 g), agar (2.5 g), and water (500 ml). Cultures were grown at room temperature (22 °C) for three weeks and then harvested for DNA extraction. The mycelial growth was scraped off of the agar, ground in liquid nitrogen using a mortar and pestle and the powder placed into two 2 ml centrifuge tubes. Then Qiagen Cell Lysis Solution (Qiagen, Inc., Valencia, CA, Cat. No. 158908), along with RNAse A and Proteinase K were added and the DNA was isolated with the Qiagen protocol as detailed in Gilmore et al. [23].

### Microsatellite marker development

We named each SSR locus ‘Cpur’ for *C. purpurea* followed by a number indicating the specific sequence the SSR was designed from (Table 2). SSR primers for *C. purpurea* were designed from the short read genomic sequence data generated by Schardl et al. [18]. The contig sequence information for 192 contigs, generated with the Roche/454 Titanium sequencer, was downloaded from NCBI. Twelve assembled sequences were pasted into MSATCOMMANDER [24], which identified 267 SSR sequences while Primer3 designed primer pairs for each SSR [25–27]. Default settings for both programs were used except that trinucleotide, tetranucleotide and pentanucleotide repeats were also included on the SSR search. An M13 sequence (TGTAAAACGACGGCCAGT) was added to the 5′ end of the forward primers to allow for an economic method of fluorescent labeling of PCR products [28]. A PIG-tail sequence, 5′-GTTT-3′, was added to the 5′ end of the reverse primer, to promote full adenylation of fragments and reduce the number of split peaks [29]. A total of 192 M13-tagged forward primers and corresponding reverse primers, along with fluorescently labeled universal M13 (−21) forward primers (WellRED D2, D3, or D4) were ordered from Integrated DNA Technologies (IDT, San Diego, CA). The primers were tested for amplification in four isolates, Cp03, Cp26, Cp32 and Cp33. Fifty-nine primer pairs were identified as potential candidates for fingerprinting and were subjected to a second round of testing with eight isolates (Cp25, Cp26, Cp27, Cp29, Cp30, Cp31, Cp32, and Cp33). Thermocycler amplification of the M13-tagged SSRs was performed with a touchdown PCR, consisting of an initial denaturing step of 94 °C for 3 m, then 10 cycles of 94 °C for 40 s, 62 °C for 45 s (decreasing the annealing temperature by 1.0 °C per cycle), and 72 °C for 45 s followed by 20 cycles of 94 °C for 40 s, 52 °C for 45 s, and 72 °C for 45 s; eight cycles of 94 °C for 40 s, 53 °C for 45 s, and 72 °C for 45 s; and a final extension of 72 °C for 30 min. The 15-μL PCR reaction mix contained: 3 μL of 5Χ GoTaq DNA Polymerase Buffer (Promega Corp., Madison, WI); 1.2 μL of 2.5 mM dNTPs; 1.2 μL of 25 mM MgCl_2_; 0.075 μL of 5 U/μL GoTaq DNA Polymerase; 0.18 μL of 10 mM forward primer; 0.75 μL of 10 mM reverse primer; 0.75 μL of 10 mM M13-fluorescent tag, WellRED D2, D3, or D4; and 1.5 μL of 3 ngμL^−1^ template DNA. Since the expected size of each amplicon was not known, an equal number of primer pairs was labeled with the different dyes, WellRED D2, D3, and D4 to allow pooling for capillary electrophoresis separation using the Beckman Coulter CEQ 8000 (Beckman Coulter, Inc., Brea CA). PCR products amplified in this first screening in the four test genotypes were then separated by 1.5 % agarose gel electrophoresis (1X TBE), and visualized with ethidium bromide to confirm amplification. PCR products from three primer pairs labeled with each of the three dyes were pooled and separated by capillary electrophoresis. The electropherograms were then scored and multiplexes were developed based on amplification and lack of fragment overlap.

### Statistical analysis

In order to infer and distinguish an optimal number of groups within the sample, discriminant analysis of principle components was performed using the R package adegenet [30–33]. The optimal number of groups was determined using the function adegenet::find.clusters. We retained 40 principle components and examined a plot of Bayesian information criteria (BIC) as a function of the number of clusters for each analysis and concluded that a group number of four minimized the BIC. These groups were then submitted to discriminant analysis using adegenet::dapc and visualized. Statistical significance was determined for these groups using the Poppr function poppr::poppr.amova [34] using one thousand replicates to determine significance. A neighbor-joining tree was created using the function poppr::bruvo.boot [35] [34] using one thousand bootstrap replicates. The dendrogram was visualized in MEGA4 [36]. PowerMarker was used to identify markers that had the greatest impact on forming CA Groups 3 and 4 and to calculate the shared allele genetic distance [37]. Shannon’s-Wiener’s index (*H*) was calculated using the following formula [38].

$$ H = - \mathop \sum \limits_{i = 1}^{n} pi\ln pi. $$
